# Effect of Polyphenol Supplementation on Post-Exercise Recovery in Adult Male Soccer Players: A Systematic Review

**DOI:** 10.3390/nu18101638

**Published:** 2026-05-21

**Authors:** Verónica Silva Díaz, Antonio Castillo-Paredes, Alexander Javier Iman Torres, Anthony Enrique Alves Vargas, Eduardo Guzmán-Muñoz, Jose Jairo Narrea Vargas

**Affiliations:** 1 Grupo de Investigación en Nutrición, Metabolismo y Ejercicio, Facultad de Ciencias de la Salud, Carrera de Nutrición y Dietética, Universidad Científica del Sur, Lima 15067, Peru; 100032025@cientifica.edu.pe; 2 Grupo AFySE, Investigación en Actividad Física y Salud Escolar, Escuela de Pedagogía en Educación Física, Facultad de Educación, Universidad de Las Américas, Santiago 8370040, Chile; acastillop85@gmail.com; 3 Departamento de Ciencia y Tecnología de Alimentos, Facultad de Industrias Alimentarias, Universidad Nacional de la Amazonía Peruana, Iquitos 16001, Peru; alexander.iman@unapiquitos.edu.pe (A.J.I.T.); anthony.alves@unapiquitos.edu.pe (A.E.A.V.); 4 Escuela de Kinesiología, Facultad de Salud, Universidad Santo Tomás, Talca 3460000, Chile; eguzmanm@santotomas.cl; 5 Escuela de Pedagogía en Educación Física, Facultad de Educación, Universidad Autónoma de Chile, Talca 3460000, Chile

**Keywords:** polyphenols, sports, oxidative stress, inflammation, athletic performance

## Abstract

**Background/Objectives**: Soccer involves high physiological demands that induce neuromuscular fatigue, muscle damage, inflammation, and oxidative stress, impairing recovery between training sessions and competitions. Polyphenols have been proposed as a nutritional strategy to modulate these responses; however, evidence in soccer players is limited and heterogeneous. This systematic review aimed to synthesize the evidence on the effects of polyphenol supplementation on post-exercise recovery in adult male soccer players. **Methods**: A systematic review was conducted following PRISMA guidelines, with a protocol registered in the Open Science Framework. Randomized controlled trials (RCTs) evaluating polyphenol supplementation versus placebo on post-exercise recovery in adult soccer players were included. Searches were performed in PubMed, Scopus, Web of Science, and the Cochrane Library up to April 2026. Risk of bias was assessed using RoB 2, and certainty of evidence using GRADE. Due to heterogeneity, a qualitative synthesis was conducted. **Results**: Eight RCTs were included. Interventions involved tart cherry juice, pomegranate juice, beetroot juice, curcumin, and tea extracts. Evidence was inconsistent for biomarkers of muscle damage, inflammation, and oxidative stress, with most studies reporting no significant differences versus placebo. In contrast, beneficial trends for perceptual outcomes, particularly reduced muscle soreness and improved subjective well-being, were mainly observed in studies using tart cherry juice, beetroot juice, and curcumin. Evidence for oxidative stress and functional recovery was limited and heterogeneous. Certainty of evidence was low for most outcomes and very low for oxidative stress and functional recovery. **Conclusions**: Polyphenol supplementation, particularly tart cherry juice, beetroot juice, and curcumin, may improve perceptual recovery in adult male soccer players, particularly by reducing muscle soreness and enhancing subjective well-being. However, evidence on physiological biomarkers and functional recovery remains inconsistent and of low certainty. Further well-designed RCTs are required to establish robust recommendations in competitive soccer.

## 1. Introduction

Soccer is one of the most widely practiced and studied sports worldwide, characterized by high physiological demands that combine intermittent high-intensity efforts, repeated sprints, frequent accelerations and decelerations, changes in direction, and incomplete recovery periods [1,2,3]. These demands impose substantial mechanical and metabolic loads, which can induce neuromuscular fatigue, muscle damage, acute inflammation, and disturbances in redox balance following training and competition [4,5]. If these processes are not adequately managed, performance in subsequent sessions may be compromised, and the risk of muscle injury may increase [6,7,8]. In this context, inadequate recovery and the accumulation of fatigue across training and competition may contribute to states of functional overreaching or, in more severe cases, overtraining syndrome, in which nutritional strategies play a key role in modulating inflammation, oxidative stress, and overall recovery capacity [9].

Post-exercise recovery is therefore a critical component of sustained performance in soccer. During congested competitive schedules, recovery strategies are essential to restore physical performance, limit residual fatigue, and maintain player availability between matches [7,8]. In this context, sports nutrition has been recognized as a practical and potentially effective tool to accelerate energy substrate replenishment, modulate muscle damage, and optimize recovery when the time between matches is limited [10,11]. Among the proposed nutritional strategies, in addition to carbohydrates and proteins, the use of bioactive compounds such as polyphenols has been suggested due to their potential role in modulating inflammatory and oxidative processes associated with fatigue and recovery [11].

Polyphenols are plant-derived bioactive compounds found in fruits, vegetables, tea, cocoa, and other foods, and include flavonoids, phenolic acids, stilbenes, and other subgroups with diverse biological properties [12,13,14]. From a structural perspective, polyphenols can be broadly classified into flavonoids (e.g., catechins and anthocyanins) and non-flavonoids (e.g., phenolic acids, stilbenes, and curcuminoids), which may differ in bioavailability and mechanisms of action [12,13]. While flavonoids have been primarily associated with the modulation of oxidative stress and inflammation, some non-flavonoid compounds may also influence vascular function and cellular signaling pathways involved in muscle recovery [15]. Although traditionally studied for their antioxidant capacity, it is now recognized that polyphenols exert their effects beyond simple free radical scavenging, including the modulation of cellular signaling pathways, inflammatory responses, and vascular function [13,14,15,16]. In the sports context, various sources of polyphenols (such as tart cherry, pomegranate, green tea, and curcumin) have been investigated for their potential to attenuate exercise-induced muscle damage and accelerate recovery [17,18,19].

However, the available evidence remains heterogeneous. A previous systematic review and meta-analysis reported that polyphenol treatments containing flavonoids may enhance muscle strength recovery and reduce muscle soreness following exercise-induced muscle damage, although no consistent effects were observed on creatine kinase levels [18]. Similarly, recent narrative reviews have highlighted promising findings but also important methodological limitations related to dosage, duration, compound characterization, and study quality [1,16,19]. Additionally, a systematic review of team-sport athletes suggested a potential beneficial effect of polyphenols on muscle recovery, although the certainty of evidence ranged from moderate to very low [20]. Nevertheless, these findings cannot be directly extrapolated to soccer, as many studies include mixed populations, different sports, or experimental protocols that do not fully replicate the specific demands of soccer match play [1,5,20].

This limitation is particularly relevant because soccer imposes specific physiological and mechanical demands, and post-exercise recovery can be assessed across different domains, including biomarkers of muscle damage, inflammation, and oxidative stress; perceptual measures such as muscle soreness and subjective well-being; and functional indicators of neuromuscular recovery [5,7,18]. Furthermore, the recent expansion of research in soccer-specific sports nutrition has identified polyphenols and recovery strategies as emerging and priority topics within the field [4]. Therefore, it is necessary to critically synthesize the available evidence specifically in adult soccer players to determine whether polyphenol supplementation provides meaningful benefits for post-exercise recovery in this population.

Accordingly, the aim of this systematic review was to synthesize the available scientific evidence on the effects of polyphenol supplementation on post-exercise recovery in adult male soccer players, considering indicators of muscle damage, inflammation, oxidative stress, muscle soreness, perceptual well-being, and, when available, functional recovery.

## 2. Materials and Methods

### 2.1. Study Design and Registration

A systematic review of the literature was conducted to evaluate the effects of polyphenol supplementation on post-exercise recovery in adult male soccer players. The study design followed the recommendations of the PRISMA statement [21], adhering to its guidelines for the identification, selection, assessment, and synthesis of scientific evidence, as well as specific recommendations for its application in sports science [22]. The checklist is provided as Supplementary File S1.

The review protocol was prospectively registered in the Open Science Framework (OSF) to ensure transparency and reproducibility of the methodological process (https://osf.io/zn4x7; accessed on 16 March 2026). The registration included the research question, eligibility criteria, search strategies, data extraction methods, and procedures for risk of bias assessment and evidence synthesis.

### 2.2. Eligibility Criteria

Eligibility criteria were defined a priori following the PICOS framework. Studies conducted in adult male soccer players aged 18–40 years were included, regardless of competitive level (amateur, collegiate, semi-professional, or professional). The restriction to male participants was defined a priori to reduce potential biological heterogeneity associated with sex-specific differences, particularly those related to hormonal fluctuations and the menstrual cycle, which may influence inflammatory responses, oxidative stress, and muscle recovery. This methodological decision was made to enhance comparability across the included studies.

Eligible studies were original randomized experimental designs (Randomized controlled trials (RCTs), including both parallel-group and crossover designs), published in English within the last 10 years up to 14 April 2026. The restriction to English-language publications and studies published within the last 10 years was applied to ensure the inclusion of contemporary evidence and the feasibility of analysis. Studies were required to evaluate polyphenol supplementation administered in isolation, either as total polyphenols or as specific classes, subclasses, or phenolic compounds (e.g., flavonoids, anthocyanins, catechins, or resveratrol). Various administration formats were accepted, including capsules, tablets, powders, liquid extracts, or standardized functional foods, provided that the supplement was clearly identified and its composition specified.

Additionally, studies were required to explicitly report the type of polyphenol or phenolic group, the administered dose, and the supplementation protocol in terms of frequency and duration. Only interventions with quantifiable doses that allowed comparison across studies were included. As a comparator, studies had to include a control or placebo group.

Regarding outcomes, studies were included if they assessed at least one indicator of post-exercise recovery, such as biomarkers of muscle damage, inflammatory markers, oxidative stress indicators, perceptual measures (e.g., muscle soreness or subjective well-being), or functional indicators related to neuromuscular performance.

Studies published in languages other than English, those without a control or placebo group, and those lacking a clear specification of the polyphenol type, dose, or supplementation protocol were excluded. Combined interventions in which polyphenols were co-administered with other nutritional supplements with potential effects on recovery (e.g., whey protein, creatine, essential amino acids, or caffeine) were also excluded due to the inability to isolate the specific effect of polyphenols. Additionally, observational studies, reviews, meta-analyses, animal studies, and studies conducted in populations other than soccer players were excluded.

### 2.3. Information Sources and Search Strategy

A systematic literature search was conducted in the electronic databases PubMed, Scopus, Cochrane Library, and Web of Science to identify relevant studies on polyphenol supplementation and its effects on post-exercise recovery in soccer players. The search strategy combined controlled vocabulary terms (MeSH) and free-text keywords using Boolean operators (AND, OR) to optimize sensitivity and specificity.

The search was conducted up to 14 April 2026, with no initial restrictions on study design during the identification phase. Subsequently, predefined eligibility criteria were applied. The search strategy was adapted to the specific characteristics of each database.

The search terms included four main components: (1) polyphenols and related compounds (e.g., polyphenols, flavonoids, resveratrol, catechins, anthocyanins, curcumin), (2) nutritional supplementation (supplementation, dietary supplements, functional foods), (3) post-exercise recovery and fatigue (recovery, muscle recovery, fatigue, post-exercise recovery), and (4) soccer (soccer, football, soccer players, football players).

The complete search strategies for each database are presented in Supplementary File S2.

### 2.4. Study Selection and Data Extraction

Following the execution of the search strategy, all retrieved references were exported to the reference manager Mendeley (Elsevier B.V., Amsterdam, The Netherlands), where duplicates were removed. The deduplicated records were then imported into Rayyan (Qatar Computing Research Institute, Doha, Qatar) to facilitate the study selection process.

Study selection was conducted in two phases. In the first phase, two independent reviewers (V.S.D. and J.J.N.V.) screened titles and abstracts to exclude studies that did not meet the eligibility criteria. In the second phase, full-text articles of potentially relevant studies were assessed using the same inclusion and exclusion criteria. Discrepancies between reviewers were resolved by consensus or, when necessary, by consultation with a third reviewer (A.C.-P.).

Gray literature was not included, as the review focused exclusively on studies published in peer-reviewed scientific journals to ensure methodological quality and validity. However, this decision may increase the risk of publication bias, which was considered when interpreting the findings.

For the included studies, two independent reviewers (V.S.D. and J.J.N.V.) performed data extraction using a standardized data extraction form developed in Microsoft Excel (Microsoft Corporation, Redmond, WA, USA). Extracted information included: author and year of publication, country, study design, participant characteristics (sample size, age, and competitive level), intervention characteristics (type of polyphenol or polyphenol group, supplement format, dose, frequency, and duration), exercise protocol, and main outcomes related to post-exercise recovery.

Outcomes of interest included biomarkers of muscle damage such as creatine kinase (CK) and lactate dehydrogenase (LDH), inflammatory markers including *C*-reactive protein (CRP) and interleukin-6 (IL-6), oxidative stress indicators such as malondialdehyde (MDA) and total antioxidant capacity (TAC), physical performance measures (e.g., countermovement jump (CMJ) and muscle strength), and perceptual variables including delayed onset muscle soreness (DOMS) and subjective well-being. When methodological details or relevant data were unclear, attempts were made to contact corresponding authors for additional information.

Due to expected heterogeneity in polyphenol types, dosages, intervention duration, and recovery assessment methods, results were synthesized using a qualitative and descriptive approach. Studies were grouped according to outcomes, type of polyphenol, and intervention characteristics. Findings were presented in tables and described narratively, considering statistical significance, direction of effect, and consistency across studies.

When multiple outcomes or time points were reported within a single study, priority was given to outcomes most directly related to post-exercise recovery, including muscle damage, inflammation, oxidative stress, perceptual variables, and functional performance. For studies reporting multiple post-exercise time points, results corresponding to the peak response or the most commonly reported time points (e.g., 24–48 h post-exercise) were prioritized to facilitate comparability across studies.

### 2.5. Risk of Bias Assessment

Risk of bias in the included studies was independently assessed by two reviewers (V.S.D. and J.J.N.V.). The Cochrane Risk of Bias tool version 2 (RoB 2) [23] was used for RCTs, applying the standard version for parallel-group studies and the adapted version for crossover trials [24].

For parallel-group studies, the following domains were assessed: (D1) bias arising from the randomization process, (D2) bias due to deviations from intended interventions, (D3) bias due to missing outcome data, (D4) bias in measurement of the outcome, and (D5) bias in selection of the reported result.

For crossover trials, an additional domain related to period and carryover effects (DS) was evaluated.

Each domain was classified as “low risk”, “some concerns”, or “high risk”, according to RoB 2 recommendations. The overall risk of bias judgment for each study was determined using the tool’s algorithms. Discrepancies were resolved by consensus or consultation with a third reviewer (A.C.-P.).

### 2.6. Data Synthesis

Due to clinical and methodological heterogeneity among the included studies, a quantitative meta-analysis was not performed. Instead, data were synthesized using a qualitative and descriptive approach.

Heterogeneity was explored qualitatively by examining variability in participant characteristics (age and competitive level), type and format of polyphenol supplementation, dose and duration of interventions, and methods used to assess post-exercise recovery.

For the synthesis, studies were grouped according to outcome type, including muscle damage, inflammation, oxidative stress, muscle soreness and perceptual variables, and functional recovery. Within each category, the direction of effect (beneficial, null, or negative), magnitude of reported outcomes, and consistency across studies were examined.

The results from individual studies were summarized in tables and described narratively, highlighting methodological differences and consistency of findings. An overall synthesis of the evidence was conducted for each outcome, considering the number of studies, proportion of favorable results, and methodological quality.

Although a meta-analysis was not conducted, planned effect measures included between-group differences, within-group changes, and reported statistical significance (*p*-values), as described in the included studies. When available, the direction of effects was also considered in the qualitative synthesis.

### 2.7. Certainty of Evidence

The certainty of evidence for the main outcomes related to post-exercise recovery was assessed using the GRADE (Grading of Recommendations Assessment, Development and Evaluation) approach [25]. This method considers five domains: risk of bias, inconsistency, indirectness, imprecision, and publication bias.

As the included studies were RCTs, the certainty of evidence was initially rated as high and subsequently downgraded based on identified limitations. The factors considered included risk of bias, heterogeneity of results, variability in interventions and populations, sample size, and precision of reported effects.

The certainty of evidence was classified as high, moderate, low, or very low, according to GRADE recommendations. The results are presented narratively and summarized in evidence tables, integrating direction of effect, number of studies, and methodological quality for each outcome.

## 3. Results

### 3.1. Study Selection

A total of 139 records were identified through database searching (PubMed, *n* = 22; Scopus, *n* = 41; Cochrane Library, *n* = 34; Web of Science, *n* = 42). After removing 52 duplicate records and 18 records for other reasons (protocol records), 69 records remained for screening.

Following title and abstract screening, 54 records were excluded, leaving 15 studies eligible for full-text assessment. All articles were retrieved and assessed in full.

Of these, 7 studies were excluded at the full-text stage for the following reasons: ineligible population (*n* = 3), non-randomized study design (*n* = 3), and combined supplementation protocols (*n* = 1).

Finally, 8 studies met the inclusion criteria and were included in the systematic review.

**Figure 1:**
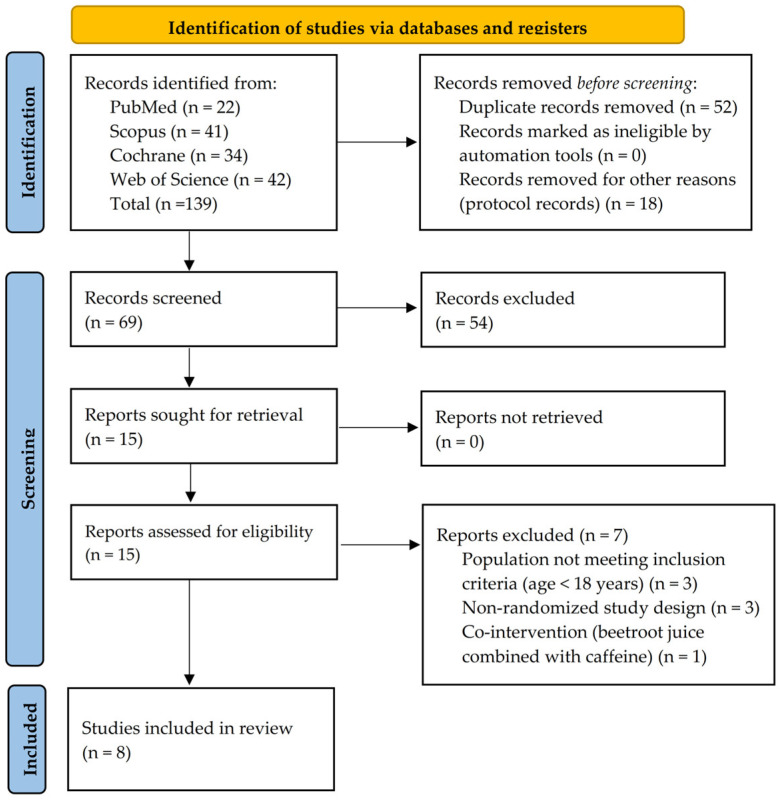
PRISMA Flow Diagram.

The study selection process is presented in Figure 1.

### 3.2. Characteristics of Included Studies

The general characteristics of the included studies are presented in Table 1. In total, eight RCTs evaluating the effects of polyphenol supplementation on post-exercise recovery in adult male soccer players were included.

The studies were conducted across different geographical regions, including Europe (United Kingdom), Asia (Japan, South Korea/China), Africa (Tunisia, Algeria), and the Middle East (Iran), reflecting a heterogeneous distribution of the studied populations. All studies included exclusively male soccer players, with sample sizes ranging from 10 to 54 participants.

Regarding participant characteristics, most studies involved young adults aged approximately 19 to 25 years. In terms of competitive level, considerable variability was observed, including professional, semi-professional, collegiate, and amateur players, which may influence the response to the evaluated interventions.

All included studies were RCTs, predominantly double-blind and placebo-controlled. Both parallel-group and crossover designs were identified, introducing differences in experimental structure and control of interindividual variability.

Regarding interventions, studies evaluated different types of polyphenols or polyphenol-rich foods, including beetroot juice, tart cherry concentrate, curcumin, pomegranate juice, and tea extracts. Dosages, administration forms, and supplementation duration showed substantial heterogeneity, with protocols ranging from short-term interventions (three days) to chronic supplementation lasting up to six weeks.

Control groups used isocaloric placebos or substances with similar appearance to the interventions, such as maltodextrin, flavored beverages, or medium-chain triglycerides, allowing adequate blinding in most studies.

Finally, regarding funding, most studies did not report this information, whereas some indicated institutional support or specific funding sources, which is relevant when considering potential sources of bias.

### 3.3. Risk of Bias

**Figure 2:**
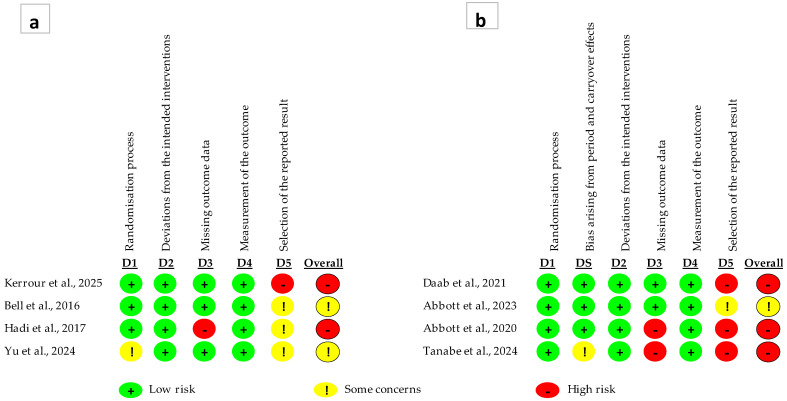
Risk of bias assessment using the RoB 2 tool: (**a**) risk of bias by domain in parallel-group studies [26,27,31,33]; (**b**) risk of bias by domain in crossover studies [28,29,30,32].

The results of the risk of bias assessment are presented in Figure 2. In parallel-group studies (Figure 2a), several studies showed low risk of bias in specific domains; however, concerns were mainly identified in the selection of reported results (D5) and, to a lesser extent, in missing outcome data (D3). Consequently, half of these studies were classified as having some concerns, while the remaining half were rated as high risk of bias due to limitations in specific domains.

In crossover studies (Figure 2b), although several studies demonstrated low risk of bias in the randomization process and outcome measurement, important limitations were identified in domains related to missing outcome data (D3) and selection of reported results (D5). Additionally, in some cases, concerns related to potential carryover effects were observed, which may compromise the internal validity of the findings.

Overall, no study was classified as having a low risk of bias across all domains. The most frequent methodological concerns were related to the selection of reported results (D5) and missing outcome data (D3), while, in crossover trials, additional concerns were observed regarding potential period and carryover effects. These limitations should be carefully considered when interpreting the findings of this review.

### 3.4. Results of the Included Studies

The results of the included studies were grouped according to the main outcomes related to post-exercise recovery, including DOMS, biomarkers of muscle damage, inflammation, oxidative stress, and functional recovery (Table 2).

#### 3.4.1. DOMS and Perceptual Variables

Muscle soreness was one of the most frequently assessed outcomes, showing heterogeneous results but with a tendency toward beneficial effects.

Studies evaluating curcumin reported contrasting findings. Abbott et al. [30] observed a significant reduction in DOMS and improved subjective well-being following exercise, whereas Tanabe et al. [32] found no significant differences between groups for any of the evaluated outcomes.

Regarding anthocyanin-rich juices (tart cherry), findings were inconsistent. Bell et al. [26] reported a significant reduction in DOMS, whereas Abbott et al. [28] found no improvements in muscle soreness or subjective well-being.

Similarly, Daab et al. [29] reported that beetroot supplementation reduced DOMS at specific time points (immediately and at 24 h), although no effects were observed on other physiological markers.

#### 3.4.2. Biomarkers of Muscle Damage, Inflammation, and Oxidative Stress

Physiological biomarkers showed a less consistent response to polyphenol supplementation.

In terms of muscle damage (creatine kinase (CK), lactate dehydrogenase (LDH), and aspartate aminotransferase (AST)), most studies reported no significant differences between groups. Bell et al. [26], Hadi et al. [27], and Daab et al. [29] found no changes in CK or other primary markers. In contrast, Kerrour et al. [33] reported a significant reduction in CK following supplementation with pomegranate and beetroot juice.

Regarding inflammation, results were limited. Bell et al. [26] reported a reduction in interleukin-6 (IL-6), whereas other studies found no changes in markers such as *C*-reactive protein (CRP).

For oxidative stress, Hadi et al. [27] reported significant improvements in malondialdehyde (MDA) and total antioxidant capacity (TAC) following supplementation with green tea and hibiscus extracts, whereas Bell et al. [26] found no differences in lipid hydroperoxides (LOOH).

#### 3.4.3. Physical Performance and Functional Recovery

Evidence on functional recovery was limited and heterogeneous. Yu et al. [31] reported that tart cherry juice supplementation attenuated the decline in post-exercise muscle performance, showing beneficial effects in strength and power tests (including countermovement jump (CMJ)), with significant group × time interactions across multiple variables. In contrast, Tanabe et al. [32] found no improvements in physical performance or functional outcomes following curcumin supplementation.

### 3.5. Synthesis of Results

The synthesis of results revealed substantial heterogeneity across the included studies in terms of the types of polyphenols evaluated, supplementation protocols, and outcomes assessed (Table 3).

Additionally, an important source of heterogeneity was the duration of supplementation protocols. Some studies evaluated acute effects (≤3 days), primarily assessing immediate post-exercise responses, whereas others investigated chronic supplementation (≥7 days), potentially allowing for cumulative physiological adaptations. These differences may partly explain inconsistencies across outcomes, particularly for physiological biomarkers. Acute protocols (e.g., Abbott et al. [28,30]; Tanabe et al. [32]) tended to show more variable effects, whereas longer supplementation periods (e.g., Hadi et al. [27]; Yu et al. [31]; Kerrour et al. [33]) were more frequently associated with favorable trends in selected outcomes.

Regarding biomarkers of muscle damage, the evidence was inconsistent, with predominantly null findings across most studies. Only one study reported favorable effects [33], suggesting that polyphenol supplementation does not have a clear impact on reducing exercise-induced muscle damage.

For inflammatory markers, the evidence was limited and heterogeneous. Although some isolated positive effects were observed [26], particularly for IL-6, these findings were not consistent across studies, with most reporting no significant differences compared with placebo [29,32,33].

Similarly, findings for oxidative stress were inconsistent. One study reported improvements in antioxidant status [27], but no clear pattern or consistency was observed across the different markers evaluated [26].

In contrast, the outcome most consistently affected was muscle soreness and perceptual fatigue variables, with most studies showing a tendency toward beneficial effects [26,29,30], although not uniformly [28,32]. This suggests that polyphenols may have a greater impact on subjective perceptions of recovery than on physiological markers.

Finally, evidence on functional recovery was very limited and heterogeneous, with inconsistent findings across the few available studies, preventing firm conclusions for this outcome.

Overall, the findings indicate that the effects of polyphenol supplementation on post-exercise recovery in adult male soccer players are inconsistent and dependent on the type of outcome assessed, with stronger evidence for benefits in perceptual variables than in physiological indicators.

When available, quantitative data such as between-group differences, statistical significance, and direction of effects were extracted and reported to enhance the interpretability of findings, although consistent reporting of effect sizes and confidence intervals was limited across studies.

Importantly, the interpretation of these findings should consider that most included studies presented some concerns or high risk of bias, particularly in the selection of reported results, missing outcome data, and, in crossover designs, potential carryover effects. These methodological limitations may partly explain the inconsistency observed across outcomes.

### 3.6. Certainty of Evidence

The certainty of evidence for the evaluated outcomes is presented in Table 3. Overall, the certainty of evidence was low for most outcomes, including muscle damage, inflammation, and muscle soreness. This classification was primarily due to methodological limitations (risk of bias) and inconsistency of results across studies.

For oxidative stress and functional recovery, the certainty of evidence was rated as very low due to additional limitations, including imprecision (small sample sizes), inconsistency, and the limited number of available studies, which reduced the reliability and generalizability of the findings.

The certainty of evidence was downgraded across outcomes based on GRADE domains. Indirectness was not considered a major concern, as all included studies involved soccer players, although variability in competitive level and intervention protocols may have contributed to heterogeneity. Potential publication bias could not be formally assessed due to the limited number of studies, but was considered when interpreting the findings.

## 4. Discussion

### 4.1. Main Findings

This systematic review aimed to synthesize the evidence on the effects of polyphenol supplementation on post-exercise recovery in adult male soccer players. Overall, the findings indicate that the available evidence is heterogeneous and limited, with a more consistent tendency toward benefits in perceptual outcomes, such as DOMS and subjective well-being, compared with physiological biomarkers of muscle damage, inflammation, or oxidative stress.

First, the results suggest that polyphenols may exert a more favorable effect on perceptual recovery than on physiological recovery. Some included studies reported reductions in muscle soreness or improvements in well-being [26,29,30], whereas others found no significant differences [28,32]. This pattern partially aligns with previous evidence in athletes and physically active individuals, where flavonoid-based polyphenols have shown modest reductions in muscle soreness but no consistent effects on creatine kinase (CK) [15]. Similarly, a systematic review of team-sport athletes concluded that polyphenols may have a beneficial impact on muscle recovery, although with certainty of evidence ranging from moderate to very low [20].

In contrast, evidence for biomarkers of muscle damage was less convincing. Most studies included in this review reported no significant changes in CK, lactate dehydrogenase (LDH), or aspartate aminotransferase (AST) following supplementation, with the exception of Kerrour et al. [33], who observed a reduction in CK following pomegranate and beetroot juice supplementation. Overall, this finding is consistent with previous meta-analyses indicating that polyphenols may improve certain functional or perceptual outcomes without necessarily reducing circulating CK levels [15]. This may be explained by the fact that CK is an indirect marker, highly variable between individuals and influenced by exercise type, training status, and timing of measurement [4,10].

Regarding inflammation, one study reported attenuation of interleukin-6 (IL-6) [26], whereas others found no clear changes in markers such as *C*-reactive protein (CRP) or high-sensitivity CRP (hs-CRP) [32,33]. Thus, the findings suggest that some polyphenols may modulate exercise-induced acute inflammation, although without a consistent response across markers. This pattern has also been reported in reviews of antioxidant supplementation in soccer, where compounds such as curcumin, tangeretin, aronia, and other antioxidants have shown isolated positive effects on IL-6, CRP, or related mediators, albeit with substantial methodological variability [16].

With respect to oxidative stress, the evidence is limited and inconsistent. Hadi et al. [27] reported reductions in malondialdehyde (MDA) and increases in total antioxidant capacity (TAC) following supplementation with green tea and hibiscus extracts, whereas Bell et al. [26] found no differences in lipid hydroperoxides (LOOH). Although these findings partially support the biological plausibility of polyphenols as modulators of redox balance, they do not allow firm conclusions. This is particularly relevant in soccer, where competitive exercise induces acute increases in oxidative stress and transient alterations in antioxidant status that may persist for several hours or days after a match [4]. However, it should also be considered that the oxidative response to exercise is not exclusively detrimental, as it plays a role in signaling and training adaptations [10,13].

Regarding functional recovery was also limited. One study reported favorable effects of tart cherry juice on countermovement jump (CMJ) and other strength measures [31], whereas another study found no improvements in CMJ or reactive strength index (RSI) following curcumin supplementation [32]. Therefore, although there is a preliminary signal of potential functional benefit, the current evidence remains insufficient to draw firm conclusions. This is consistent with previous reviews suggesting that polyphenols may enhance muscle strength recovery in certain contexts, although results depend largely on the type of compound, dose, supplementation duration, and exercise model used [15,17,18].

This apparent discrepancy between perceptual and physiological outcomes may be explained by the complex nature of recovery. Perceptual responses such as muscle soreness and well-being are influenced not only by local muscle damage or inflammation but also by contextual factors, including training load, nutritional status, sleep quality, baseline antioxidant intake, competitive level, and timing of supplementation. These factors may contribute to more detectable changes in subjective outcomes, even in the absence of consistent alterations in classical biochemical markers.

This observation may be particularly relevant in the context of accumulated fatigue and insufficient recovery, where nutritional strategies have been proposed as modulators of systemic stress responses, including inflammation and oxidative stress, which are involved in the development of overreaching and overtraining states [9].

Overall, the findings of this review suggest that polyphenols may be more effective in attenuating subjective symptoms of fatigue and muscle soreness than in consistently modifying classical biochemical markers of recovery. This aligns with the current understanding of recovery as a multifactorial process integrating physiological, functional, and perceptual responses [8,34].

However, these findings should be interpreted with caution, as no study was rated as low risk of bias across all domains, and recurrent methodological limitations—particularly selective reporting, missing data, and potential carryover effects—may have influenced the observed results.

### 4.2. Heterogeneity of Interventions

One of the main challenges in interpreting the available evidence was the marked heterogeneity across interventions, both in the nature of the bioactive compounds and in supplementation protocols and measured outcomes.

First, the included studies evaluated different sources of polyphenols with distinct phytochemical profiles and potentially different mechanisms of action. At least four main groups can be identified: anthocyanins (tart cherry and pomegranate), curcuminoids (curcumin), flavonoids (mainly catechins and anthocyanins in green tea and hibiscus), and nitrate-rich sources containing phenolic compounds (e.g., beetroot). This diversity is relevant because polyphenols do not constitute a homogeneous class of compounds; they differ in chemical structure, bioavailability, metabolism, and their capacity to interact with antioxidant, inflammatory, and vascular pathways [12,13,35]. Therefore, their effects on recovery are unlikely to be equivalent.

Second, there was substantial variability in dose and duration of supplementation. Some studies employed short-term protocols of 2–3 days [28,30,32], whereas others used longer interventions, such as 3 weeks [31], 22 days [33], or 6 weeks [27]. This difference may be critical, as previous reviews suggest that the effects of polyphenols on muscle recovery are more likely when intake is sustained for several days before and after exercise, particularly at relatively high doses [15,17]. Similarly, a review of team-sport athletes proposed that doses around 60 mL/day (divided into two servings) for more than 7 days may improve functional recovery and muscle soreness [20]. In contrast, short-duration or poorly characterized interventions may underestimate the true effect.

Heterogeneity was also observed in the exercise models used. Some studies reproduced soccer-specific loads through simulated or official matches, whereas others used intermittent or resistance-based protocols. This is relevant because soccer involves intermittent high-intensity efforts, repeated sprints, accelerations, decelerations, changes in direction, and accumulated eccentric load [1,2]. Not all experimental protocols replicate these demands with the same ecological validity.

Another important aspect was the diversity of recovery outcomes assessed. While some studies focused on biochemical markers, others prioritized perceptual or functional variables. This diversity limits comparability and may partly explain apparent inconsistencies. For example, a polyphenol may reduce muscle soreness without affecting CK, or improve functional performance without altering CRP. This is consistent with previous reviews emphasizing that recovery should not be interpreted based on a single marker but rather through a multidimensional approach [8,34].

Finally, heterogeneity related to participants’ competitive level should be considered. This review included amateur, collegiate, semi-professional, and professional players. Differences in training status, chronic adaptation, nutritional status, and competitive load may influence responses to supplementation. In elite soccer, where training and match demands require individualized monitoring and recovery strategies, these differences may be particularly relevant [6,7,11]. Therefore, extrapolation across competitive levels should be made with caution.

### 4.3. Strengths and Limitations

A key strength of this review is its specific focus on adult male soccer players, a population in which polyphenol supplementation has been less systematically synthesized compared to other sports contexts. This specificity is important given the unique physiological demands and competitive schedules in soccer, where rapid recovery between sessions or matches may be critical for performance and player availability [8,11]. Additionally, only RCTs were included, risk of bias was assessed using RoB 2, and certainty of evidence was evaluated using the GRADE approach, enhancing methodological rigor.

Another strength was the integrated consideration of multiple recovery domains, including muscle damage, inflammation, oxidative stress, muscle soreness, and functional recovery. This allowed for a broader interpretation consistent with the multifactorial nature of recovery [34].

However, several limitations should be acknowledged. First, the number of included studies was small, and several trials had limited sample sizes, reducing the precision of findings. Second, most studies presented some concerns or high risk of bias, particularly in the selection of reported outcomes, missing data, and, in crossover designs, potential period or carryover effects. These methodological limitations contributed to the low or very low certainty of evidence and are consistent with the GRADE assessment, in which most outcomes were downgraded due to risk of bias, inconsistency, and imprecision.

Another relevant limitation relates to the assessment of perceptual outcomes, such as muscle soreness and subjective well-being, which are inherently susceptible to placebo effects and reporting bias. In nutritional interventions using juices or functional foods, achieving effective double-blinding may be challenging due to their distinctive organoleptic properties (e.g., taste, color, and texture). These characteristics may allow participants to identify the intervention, potentially influencing subjective responses and contributing to the observed beneficial effects in perceptual outcomes.

Additionally, there was substantial clinical and methodological heterogeneity, including differences in polyphenol type, dose, duration, participant characteristics, and outcome measures, which precluded quantitative meta-analysis. Moreover, all studies were conducted in male soccer players, limiting generalizability to female athletes. This is particularly relevant given that nutritional recommendations in female players also emphasize recovery optimization and highlight existing sex-specific evidence gaps [36].

Another important limitation is that some studies did not comprehensively characterize the phenolic composition of the interventions. This is critical, as lack of analytical standardization remains a major challenge for comparing studies and establishing dose–response relationships [35]. Furthermore, polyphenol bioavailability may vary considerably depending on food matrix, processing, and interactions with other nutrients [12,13].

### 4.4. Practical and Research Implications

From a practical perspective, the findings of this review suggest that polyphenol supplementation may be considered a complementary strategy within the recovery process in adult male soccer players, particularly for its potential effects on muscle soreness and subjective recovery perception. However, current evidence does not consistently support its use for improving biomarkers of muscle damage, inflammation, or oxidative stress. Therefore, its implementation should be framed within a comprehensive recovery approach that includes adequate carbohydrate and protein intake, hydration, sleep, and load management [8,11].

In congested competitive schedules, where recovery time between matches is limited, nutrition plays a key role in maintaining performance and player availability [11]. In this context, polyphenols may have situational utility, although the low certainty of evidence requires cautious interpretation and individualized application based on competitive context, compound type, and athlete tolerance.

From a research perspective, further RCTs with greater methodological rigor, larger sample sizes, and standardized protocols are needed. In particular, improved analytical characterization of polyphenols, including composition, dose, and bioavailability, is essential [12,35]. Standardization of recovery outcomes would also facilitate comparability across studies [8,34]. Additionally, future research should include different competitive levels and female soccer players, who remain underrepresented [20,36]. Finally, future studies should explore whether specific polyphenol profiles exert differential effects on distinct recovery outcomes, as this represents an emerging and priority area in sports nutrition research applied to soccer [37].

Although polyphenols exhibit biological mechanisms that support their potential role in modulating oxidative stress, inflammation, and recovery processes, biological plausibility alone does not replace the need for consistent clinical evidence and methodological robustness. Therefore, current findings should be interpreted with caution, particularly in light of the heterogeneity and limitations identified across studies.

## 5. Conclusions

Current evidence suggests that polyphenol supplementation may represent a promising nutritional strategy to support recovery in adult male soccer players, particularly in reducing muscle soreness and improving perceptual well-being. However, evidence on physiological biomarkers and functional recovery remains inconsistent and of low certainty.

These findings should be interpreted cautiously, as no included study was classified as having a low risk of bias across all domains, and common methodological limitations—particularly selective reporting, missing outcome data, and potential carryover effects—may have influenced the results. This underscores the need for more robust and standardized studies to establish their application in competitive soccer.

## Figures and Tables

**Table 1:** General Characteristics of the Included Studies.

Author and Year	Country	Sample (*n*; Sex)	Age, Body Mass, and Height	Competitive Level/Playing Experience	Study Design	Supplementation Dose and Protocol	Placebo/Control	Funding
Bell et al., 2016 [26]	United Kingdom	16 male soccer players	25 ± 4 years; 81.9 ± 6.6 kg; 180.8 ± 7.4 cm	Semi-professional	RCT, double-blind, placebo-controlled, parallel-group	Montmorency tart cherry concentrate: 30 mL, twice daily for 7 days (4 days pre + match day + post days); diluted in 100 mL water	Isocaloric placebo drink (commercial syrup with <5% fruit, maltodextrin, and water)	Not reported
Hadi et al., 2017 [27]	Iran	54 male soccer players	~21 years; 74 ± 8.6 kg; 180 ± 6 cm	Collegiate (≥4 years experience)	RCT, double-blind, placebo-controlled, parallel-group	450 mg/day green tea extract (catechin-rich) or hibiscus tea (anthocyanin-rich) for 6 weeks	Maltodextrin (identical capsules)	Funded by Isfahan University of Medical Sciences
Abbott et al., 2020 [28]	United Kingdom	10 male soccer players	19 ± 1 years; 77.3 ± 6.4 kg; 1.80 ± 0.06 m	Professional (Premier League reserve team)	RCT, double-blind, crossover, placebo-controlled	Tart cherry concentrate (30 mL diluted to 250 mL, twice daily) from match day to 36 h post-match (~3 days)	Cherry-flavored isocaloric drink (sucrose), no cherry concentrate	Not reported (supplement provided by Healthspan without influence on the study)
Daab et al., 2021 [29]	Tunisia	13 male soccer players	22.1 ± 0.5 years; 75.8 ± 5.5 kg; 178 ± 1.1 cm	Semi-professional (~7 years experience)	RCT, double-blind, crossover, placebo-controlled	Beetroot juice (150 mL, twice daily) for 7 days (3 days pre + test day + 3 days post); 250 mg/day nitrate	Isocaloric placebo (maltodextrin + protein + water, matched for volume and nutrients)	Not reported
Abbott et al., 2023 [30]	United Kingdom	11 male soccer players	19 ± 1 years; 79.4 ± 7.9 kg; 180.8 ± 5.7 cm	Professional (U-23 Premier League)	RCT, double-blind, crossover, placebo-controlled	Curcumin: 500 mg/day administered immediately post-match and at 12 and 36 h post-match (3 days)	Medium-chain triglycerides (1000 mg/day), matched appearance	Not reported
Yu et al., 2024 [31]	South Korea/China	24 male soccer players	22 ± 3 years; 75.9 ± 5.3 kg; 179.8 ± 6.4 cm	Collegiate	RCT, placebo-controlled, parallel-group	Tart cherry juice: 70 mL, twice daily for 3 weeks, followed by resisted sled training in week 4	Isocaloric placebo drink (purified water with artificial fruit components)	Not reported
Tanabe et al., 2024 [32]	Japan	20 male soccer players	20 ± 1 years; 65.6 ± 5.0 kg; 171.5 ± 5.7 cm	Collegiate (training 6 times/week)	RCT, double-blind, crossover, placebo-controlled	Curcumin: 450 mg total (90 mg × 5 doses), administered 1 h pre-match and during 48 h post-match; two matches separated by 1 week	Equivalent maltose	Financiado por JSPS KAKENHI (Research Fellowships)
Kerrour et al., 2025 [33]	Algeria	18 male soccer players	24.5 ± 5.2 years; 76.1 ± 14.2 kg; 180.0 ± 0.5 cm	Amateur	RCT, three parallel groups, placebo-controlled	Supplementation for 22 days: pomegranate juice (600 mL/day) or beetroot juice (300 mL/day)	Water beverage with artificial coloring	Not reported

**Table 2:** Main Findings of the Included Studies on the Effects of Polyphenols on Post-Exercise Recovery in Adult Male Soccer Players.

Author and Year	Post-Exercise Recovery Outcomes Assessed	Test/Measurement	Main Result (Polyphenols vs. Placebo/Control)	*p*-Value/Statistical Significance	Conclusion
Bell et al., 2016 [26]	Muscle damage: CK. Inflammation: IL-6, IL-8, TNF-α, hs-CRP, IL-1β. Oxidative stress: LOOH. Muscle soreness: DOMS.	Adapted LIST + serial blood samples + visual analog scale for DOMS	Tart cherry concentrate attenuated IL-6 response and reduced DOMS during recovery. No significant differences in CK or LOOH vs. placebo.	IL-6 and DOMS: *p* < 0.05; CK and LOOH: *p* > 0.05	Tart cherry showed benefits mainly in acute inflammation and muscle soreness, but not in classical biomarkers of muscle damage or oxidative stress.
Hadi et al., 2017 [27]	Muscle damage: AST, CK, LDH. Oxidative stress: MDA, TAC.	Fasting blood samples at baseline and after 6 weeks	Green tea and hibiscus extracts reduced MDA; hibiscus also increased TAC. No significant changes in CK or other primary muscle damage markers.	MDA: *p* < 0.01; TAC: *p* < 0.05; CK: *p* > 0.05	Green tea and hibiscus polyphenols improved oxidative status but did not clearly affect muscle damage.
Abbott et al., 2020 [28]	Muscle soreness. Fatigue/well-being: subjective well-being.	Official match + visual analog scale + well-being questionnaire	Tart cherry juice showed no differences vs. placebo in muscle soreness or subjective well-being during post-match recovery.	Muscle soreness: *p* > 0.05; well-being: *p* > 0.05	Acute tart cherry supplementation did not improve perceptual recovery in professional soccer players.
Daab et al., 2021 [29]	Muscle damage: CK, LDH. Inflammation: CRP. Muscle soreness: DOMS.	LIST + blood markers + soreness scale (0–72 h)	Beetroot juice reduced DOMS immediately and at 24 h, but did not affect CK, LDH, or CRP vs. placebo.	DOMS: *p* < 0.05 at specific time points; CK, LDH, CRP: *p* > 0.05	Beetroot juice improved muscle soreness but showed no effect on muscle damage or inflammation biomarkers.
Abbott et al., 2023 [30]	Muscle soreness: DOMS. Well-being/fatigue: subjective well-being.	90 min match + DOMS scale (0–200 mm) + well-being questionnaire	Curcumin reduced DOMS at all post-match time points and improved subjective well-being vs. control. No blood biomarkers assessed.	DOMS: *p* < 0.01; well-being: significant improvements at specific time points	Curcumin improved perceptual recovery by reducing soreness and enhancing well-being.
Yu et al., 2024 [31]	Functional performance: CMJ, hamstring and quadriceps strength	CMJ, strength tests (seated and prone knee flexion at 90°), Nordic hamstring test, RST	Tart cherry supplementation attenuated post-exercise decline in muscle performance and enhanced recovery vs. placebo across several strength and power tests.	Significant group × time interactions (*p* < 0.05) for CMJ and strength tests; some variables non-significant	Tart cherry may improve functional recovery and muscle performance following intermittent exercise.
Tanabe et al., 2024 [32]	Muscle damage: CK, U-titin. Inflammation: hs-CRP. Muscle soreness: DOMS. Performance: CMJ, RSI.	Serum biomarkers (CK, hs-CRP), ELISA (U-titin), visual analog scale (DOMS), jump tests (CMJ, RSI)	No significant differences between curcumin and placebo for any outcome following the match.	*p* > 0.05 for all comparisons	Curcumin supplementation did not improve recovery or physical performance in soccer players.
Kerrour et al., 2025 [33]	Muscle damage: CK. Inflammation: CRP.	Blood sampling pre and post High-Intensity Training session at three time points (baseline, day 12, day 22)	Both pomegranate and beetroot juice reduced CK increase vs. placebo after 22 days. No differences in CRP. No differences between interventions.	CK: *p* < 0.001 vs. placebo; CRP: *p* > 0.05	Chronic supplementation with pomegranate and beetroot juice improved biochemical muscle damage but not systemic inflammation.

Abbreviations: CK, creatine kinase; IL-6, interleukin-6; IL-8, interleukin-8; TNF-α, tumor necrosis factor alpha; hs-CRP, high-sensitivity *C*-reactive protein; IL-1β, interleukin-1 beta; AST, aspartate aminotransferase; LDH, lactate dehydrogenase; MDA, malondialdehyde; TAC, total antioxidant capacity; LOOH, lipid hydroperoxides; CRP, *C*-reactive protein; DOMS, delayed onset muscle soreness; CMJ, countermovement jump; RSI, reactive strength index; RST, repeated sprint test; LIST, Loughborough Intermittent Shuttle Test; ELISA, enzyme-linked immunosorbent assay.

**Table 3:** Overall Synthesis of Evidence and Methodological Quality.

Post-Exercise Recovery Outcome	Number of Studies	Positive Effect	Null/Negative Effect	Conclusion	Overall Certainty (GRADE)	Studies
Muscle damage (CK, LDH, AST)	5	1	4	Inconsistent evidence. Most studies do not show a significant reduction in muscle damage following polyphenol supplementation.	Low	[26,27,29,32,33]
Inflammation (CRP, IL-6, TNF-α)	4	1	3	Inconsistent evidence. Null findings predominate for inflammatory markers, with isolated favorable effects (IL-6) not consistent across studies.	Low	[26,29,32,33]
Oxidative stress (MDA, TAC, LOOH)	2	1	1	Limited and heterogeneous evidence. Some studies report improvements in antioxidant status, but without consistency across markers.	Very low	[26,27]
Muscle soreness/fatigue (DOMS, subjective well-being)	5	3	2	Heterogeneous evidence with a tendency toward beneficial effects. Most studies report reductions in muscle soreness and/or improvements in perceptual well-being, although findings are not consistent across all studies.	Low	[26,28,29,30,32]
Functional recovery (CMJ, strength, power))	2	1	1	Limited and heterogeneous evidence, with inconsistent findings across studies.	Very low	[31,32]

Abbreviations: CK, creatine kinase; LDH, lactate dehydrogenase; AST, aspartate aminotransferase; CRP, *C*-reactive protein; IL-6, interleukin-6; TNF-α, tumor necrosis factor alpha; MDA, malondialdehyde; TAC, total antioxidant capacity; LOOH, lipid hydroperoxides; DOMS, delayed onset muscle soreness; CMJ, countermovement jump.

## Data Availability

All the included studies are presented in the article.

## Supplementary Materials

The following supporting information can be downloaded at: https://www.mdpi.com/article/10.3390/nu18101638/s1, File S1: PRISMA checklist; File S2: Search strategy for each database.

## Abbreviations

The following abbreviations are used in this manuscript:

RCT	Randomized controlled trial
AST	Aspartate aminotransferase
DOMS	Delayed onset muscle soreness
CK	Creatine kinase
CMJ	Countermovement jump
CRP	*C*-reactive protein
ELISA	Enzyme-linked immunosorbent assay
GRADE	Grading of Recommendations Assessment, Development and Evaluation
hs-CRP	High-sensitivity *C*-reactive protein
IL-6	Interleukin-6
TNF-α	Tumor necrosis factor-alpha
U-titin	Urinary titin
LDH	Lactate dehydrogenase
LIST	Loughborough Intermittent Shuttle Test
LOOH	lipid hydroperoxides
MDA	malondialdehyde
OSF	Open Science Framework
PICOS	Population, Intervention, Comparator, Outcomes, and Study design
PRISMA	Preferred Reporting Items for Systematic Reviews and Meta-Analyses
RoB 2	Risk of Bias 2
RSI	Reactive strength index
RST	Repeated sprint test
TAC	Total antioxidant capacity

## References

[1] Rampinini E., Impellizzeri F.M., Castagna C., Coutts A.J., Wisløff U. (2021). Technical performance during soccer matches of the Italian Serie A league: Effect of fatigue and competitive level. J. Sci. Med. Sport.

[2] Harper D.J., Carling C., Kiely J. (2019). High-intensity acceleration and deceleration demands in elite team sports competitive match play: A systematic review and meta-analysis of Observational Studies. Sports Med..

[3] Ekstrand J., Spreco A., Bengtsson H., Bahr R. (2021). Injury rates decreased in men’s professional football: An 18-year prospective cohort study of almost 12,000 injuries sustained during 1.8 million hours of play. Br. J. Sports Med..

[4] Fatouros I.G., Chatzinikolaou A., Douroudos I.I., Nikolaidis M.G., Kyparos A., Margonis K., Michailidis Y., Vantarakis A., Taxildaris K., Katrabasas I. (2010). Time-course of changes in oxidative stress and antioxidant status responses following a soccer game. J. Strength Cond. Res..

[5] Silva J.R., Brito J., Akenhead R., Nassis G.P. (2016). The transition period in soccer: A window of opportunity. Sports Med..

[6] Ammann L., Altmann S. (2023). Training and match load ratios in professional soccer—Should we use player- or position-specific match reference values?. Front. Sports Act. Living.

[7] Akenhead R., Nassis G.P. (2016). Training load and player monitoring in high-level football: Current practice and perceptions. Int. J. Sports Physiol. Perform..

[8] Nédélec M., McCall A., Carling C., Legall F., Berthoin S., Dupont G. (2013). Recovery in soccer: Part II—Recovery strategies. Sports Med..

[9] la Torre M.E., Monda A., Messina A., de Stefano M.I., Monda V., Moscatelli F., Tafuri F., Saraiello E., Latino F., Monda M. (2023). The Potential Role of Nutrition in Overtraining Syndrome: A Narrative Review. Nutrients.

[10] Pingitore A., Lima G.P., Mastorci F., Quinones A., Iervasi G., Vassalle C. (2015). Exercise and oxidative stress: Potential effects of antioxidant dietary strategies in sports. Nutrition.

[11] Ranchordas M.K., Dawson J.T., Russell M. (2017). Practical nutritional recovery strategies for elite soccer players when limited time separates repeated matches. J. Int. Soc. Sports Nutr..

[12] El-Saadony M.T., Yang T., Saad A.M., Alkafaas S.S., Elkafas S.S., Eldeeb G.S., Mohammed D.M., Salem H.M., Korma S.A., Loutfy S.A. (2024). Polyphenols: Chemistry, bioavailability, bioactivity, nutritional aspects and human health benefits: A review. Int. J. Biol. Macromol..

[13] Scalbert A., Johnson I.T., Saltmarsh M. (2005). Polyphenols: Antioxidants and beyond. Am. J. Clin. Nutr..

[14] Valder S., Habersatter E., Kostov T., Quenzer S., Herzig L., von Bernuth J., Matits L., Herdegen V., Diel P., Isenmann E. (2024). The influence of a polyphenol-rich red berry fruit juice on recovery process and leg strength capacity after six days of intensive endurance exercise in recreational endurance athletes. Nutrients.

[15] Carey C.C., Lucey A., Doyle L. (2021). Flavonoid-containing polyphenol consumption and recovery from exercise-induced muscle damage: A systematic review and meta-analysis. Sports Med..

[16] Poulios A., Papanikolaou K., Draganidis D., Tsimeas P., Chatzinikolaou A., Tsiokanos A., Jamurtas A.Z., Fatouros I.G. (2024). The effects of antioxidant supplementation on soccer performance and recovery: A critical review of the available evidence. Nutrients.

[17] Bowtell J.L., Kelly V. (2019). Fruit-derived polyphenol supplementation for athlete recovery and performance. Sports Med..

[18] Zhang X., Zhong Y., Rajabi S. (2025). Polyphenols and post-exercise muscle damage: A comprehensive review of literature. Eur. J. Med. Res..

[19] Ortega D.G., Coburn J.W., Galpin A.J., Costa P.B. (2023). Effects of a Tart Cherry Supplement on Recovery from Exhaustive Exercise. J. Funct. Morphol. Kinesiol..

[20] Sánchez Díaz M., Martín-Castellanos A., Fernández-Elías V.E., López Torres O., Lorenzo Calvo J. (2022). Effects of polyphenol consumption on recovery in team sport athletes of both sexes: A systematic review. Nutrients.

[21] Page M.J., McKenzie J.E., Bossuyt P.M., Boutron I., Hoffmann T.C., Mulrow C.D., Shamseer L., Tetzlaff J.M., Akl E.A., Brennan S.E. (2021). The PRISMA 2020 statement: An updated guideline for reporting systematic reviews. BMJ.

[22] Ardern C.L., Büttner F., Andrade R., Weir A., Ashe M.C., Sinead H., Impellizzeri F.M., Delahunt E., Dijkstra H.P., Mathieson S. (2022). Implementing the 27-item PRISMA 2020 statement for systematic reviews in sports and exercise medicine: The PERSiST guideline. Br. J. Sports Med..

[23] Sterne J.A.C., Savović J., Page M.J., Elbers R.G., Blencowe N.S., Boutron I., Cates C.J., Cheng H.Y., Corbett M.S., Eldridge S.M. (2019). RoB 2: A revised tool for assessing risk of bias in randomised trials. BMJ.

[24] Higgins J.P.T., Thomas J., Chandler J., Cumpston M., Li T., Page M.J., Welch V. (2024). Cochrane Handbook for Systematic Reviews of Interventions, Version 6.5.

[25] Guyatt G.H., Oxman A.D., Vist G.E., Kunz R., Falck-Ytter Y., Alonso-Coello P., Schünemann H.J. (2008). GRADE: An emerging consensus on rating quality of evidence and strength of recommendations. BMJ.

[26] Bell P.G., Stevenson E., Davison G.W., Howatson G. (2016). The effects of Montmorency tart cherry concentrate supplementation on recovery following prolonged, intermittent exercise. Nutrients.

[27] Hadi A., Pourmasoumi M., Kafeshani M., Karimian J., Maracy M.R., Entezari M.H. (2017). The effect of green tea and sour tea (*Hibiscus sabdariffa* L.) supplementation on oxidative stress and muscle damage in athletes. J. Diet. Suppl..

[28] Abbott W., Brashill C., Brett A., Clifford T. (2020). Tart cherry juice: No effect on muscle function loss or muscle soreness in professional soccer players after a match. Int. J. Sports Physiol. Perform..

[29] Daab W., Bouzid M.A., Lajri M., Bouchiba M., Saafi M.A., Rebai H. (2021). Chronic beetroot juice supplementation accelerates recovery kinetics following simulated match play in soccer players. J. Am. Coll. Nutr..

[30] Abbott W., Hansell E.J., Brett A., Škarabot J., James L.J., Clifford T. (2023). Curcumin attenuates delayed-onset muscle soreness and muscle function deficits following a soccer match in male professional soccer players. Int. J. Sports Physiol. Perform..

[31] Yu T., Dong K., Jin L. (2024). Effect of tart cherry juice supplement on lower extremity strength recovery performance after periodized resisted sled-based training. J. Men’s Health.

[32] Tanabe Y., Kondo E., Sagayama H., Shimizu K., Yasumatsu M., Nakamura D., Fujii N., Takahashi H. (2024). Impact of curcumin supplementation on exercise performance and muscle damage after a soccer match: A double-blind placebo-controlled cross-over study. Eur. J. Appl. Physiol..

[33] Kerrour N.S., Guendouze N., Remini H., Lakhdara N., Zaabar S. (2025). Effects of beetroot and pomegranate juice supplementation on creatine kinase and C-reactive protein in amateur soccer players. N. Afr. J. Food Nutr. Res..

[34] Kellmann M., Bertollo M., Bosquet L., Brink M., Coutts A.J., Duffield R., Erlacher D., Halson S.L., Hecksteden A., Heidari J. (2018). Recovery and performance in sport: Consensus statement. Int. J. Sports Physiol. Perform..

[35] Lisjak M., Špoljarević M., Ravlić J., Lončarić Z., Galić L. (2026). Phenolic Compounds and Antioxidant Activity: Analytical Methods and Current Knowledge—A Review. Methods Protoc..

[36] De Sousa M.V., Lundsgaard A.M., Christensen P.M., Christensen L., Randers M.B., Mohr M., Nybo L., Kiens B., Fritzen A.M. (2022). Nutritional optimization for female elite football players: Topical review. Scand. J. Med. Sci. Sports.

[37] De Oliveira D.M., Silva A.K.A.C., Macedo A.G., Fernandes M.B., Fernandes E.V. (2025). Global research trends in sports nutrition and football over the last 20 years (2004–2024). Sports.

