# Supplementary material for: Effect of Polyphenol Supplementation on Post-Exercise Recovery in Adult Male Soccer Players: A Systematic Review
Source: Nutrients. 2026 May 21;18(10):1638. doi: 10.3390/nu18101638 (PMC13209340; doi:10.3390/nu18101638)
Supplement: Supplementary file 1 [file nutrients-18-01638-s001.zip › Supplementary File S2_Search strategy for each database.pdf]

Search strategy for each database

Database	Search strategy
Pubmed	("Polyphenols"[Mesh] OR polyphenol*[tiab] OR "Flavonoids"[Mesh] OR flavonoid*[tiab] OR "Resveratrol"[Mesh] OR resveratrol[tiab] OR quercetin[tiab] OR "Green Tea"[Mesh] OR "green tea"[tiab] OR catechin*[tiab] OR anthocyanin*[tiab] OR "Grape Seed Extract"[tiab] OR curcumin[tiab] OR "Curcumin"[Mesh] OR "cherry juice"[tiab] OR "tart cherry juice"[tiab] OR "beetroot juice"[tiab] OR "beet juice"[tiab] OR "beet root juice"[tiab] OR cacao[tiab] OR cocoa[tiab] OR "dark chocolate"[tiab] OR chocolate[tiab]) AND ("Dietary Supplements"[Mesh] OR supplement*[tiab] OR "Supplementation"[tiab] OR "functional food"[tiab] OR "functional foods"[Mesh]) AND ("Recovery of Function"[Mesh] OR "Fatigue"[Mesh] OR recovery[tiab] OR "post-exercise recovery"[tiab] OR "muscle recovery"[tiab] OR "exercise recovery"[tiab] OR "recovery time"[tiab] OR "fatigue recovery"[tiab]) AND ("Soccer"[Mesh] OR football[tiab] OR soccer[tiab] OR "soccer player"[tiab] OR "football player"[tiab])
Scopus	(polyphenol* OR flavonoid* OR resveratrol OR quercetin OR green tea OR catechin* OR anthocyanin* OR "grape seed extract" OR curcumin OR "cherry juice" OR "tart cherry juice" OR "beetroot juice" OR "beet juice" OR cacao OR cocoa OR "dark chocolate" OR chocolate) AND (supplement* OR supplementation OR "functional food" OR "functional foods") AND (recovery OR "post exercise recovery" OR "muscle recovery" OR "exercise recovery" OR "recovery time" OR fatigue OR "fatigue recovery") AND (soccer OR football OR "soccer player" OR "football player")
Chochrane	polyphenol* OR flavonoid* OR resveratrol OR quercetin OR "green tea" OR catechin* OR anthocyanin* OR "grape seed extract" OR curcumin OR "cherry juice" OR "tart cherry juice" OR "beetroot juice" OR cacao OR cocoa OR chocolate<br>AND supplement OR supplementation OR "functional food" OR "functional

	foods" AND recovery OR "post-exercise recovery" OR "muscle recovery" OR "exercise recovery" OR fatigue AND soccer OR football OR "soccer player" OR "football player"
Web of Science	TS=(polyphenol* OR flavonoid* OR resveratrol OR quercetin OR "green tea" OR catechin* OR anthocyanin* OR "grape seed extract" OR curcumin OR "cherry juice" OR "tart cherry juice" OR "beetroot juice" OR "beet juice" OR cacao OR cocoa OR "dark chocolate" OR chocolate)<br>AND TS=(supplement* OR supplementation OR "functional food" OR "functional foods")<br>AND TS=(recovery OR "post exercise recovery" OR "muscle recovery" OR "exercise recovery" OR "recovery time" OR fatigue OR "fatigue recovery")<br>AND TS=(soccer OR football OR "soccer player" OR "football player")
